# Effects of histones related to sperm chromatin on embryo development and ART outcomes

**DOI:** 10.1097/MD.0000000000036113

**Published:** 2023-11-24

**Authors:** Mingyue Wang, Haibo Zhu, Yuting Jiang, Ruizhi Liu, Ruixue Wang

**Affiliations:** a Center of Reproductive Medicine and Center of Prenatal Diagnosis, First Hospital, Jilin University, Changchun, China.

**Keywords:** assisted reproductive technology, embryonic development, pregnancy outcomes, sperm histones

## Abstract

In the process of spermatogenesis and maturation, histones of the sperm nucleus were gradually replaced by protamine. Abnormal sperm nucleoprotein histotype conversion can make sperm DNA unstable and affect sperm function. The aim of this study is to investigate the impact of high and low proportion of sperm histone positivity in semen sample on embryonic development and assisted reproductive technology results, and to evaluate its diagnostic value in assisted reproduction. Sperm nuclear status was detected with aniline blue staining. Under acidic conditions, aniline blue combines with histones rich in lysine residues to form blue compounds. The groups were divided according to the critical value of sperm histone positive ratio of 30%. Using the intracytoplasmic sperm injection procedure, the fertilization rate and normal fertilization rate in the normal sperm histone positive ratio group were significantly higher than those in the abnormal group, and the difference was statistically significant (*P* < .001). Using the in vitro fertilization procedure, the effect of sperm histone positive ratio on each index was not statistically different. Overall the study provides some preliminary evidence that abnormal sperm histones may be a factor that affects the fertilization success of intracytoplasmic sperm injection procedures. However, more research is needed to confirm this finding to determine the exact mechanism by which abnormal sperm histones affect fertilization.

## 1. Introduction

According to statistics, 8% to 12% of couples worldwide suffer from infertility. Only male factors account for about 20% of infertile couples, and another 30% to 40% of infertile couples are also related to male factors.^[1]^ In recent years, the incidence of infertility caused by male factors has increased year by year. The factors affecting male sperm quality are diverse.^[2,3]^

During spermatogenesis, the number of chromosomes and DNA content changes, as does the nuclear protein that binds to DNA. Chromatin remodeling is an unusual and important step in this process. The basic protein bound to sperm and nuclear DNA undergoes a histones switch, and histones rich in lysine residues are gradually replaced by protamine rich in arginine and cysteine residues.^[4]^ This is an important sign of sperm nuclear maturation and an important basis for the transmission of genetic material.^[5,6]^ After spermatogenesis, the chromatin of mature sperm has a ring structure that is tightly compacted and resistant to denaturation.^[7]^ The abnormal expression of sperm-specific nuclear proteins or their association with chromatin can alter sperm chromatin structure and affect sperm function.^[8,9]^ In the 6th edition of the WHO manual for human semen analysis, sperm DNA fragmentation, sperm chromatin, sperm aneuploidy, and semen interleukin have been added.

Genes encode necessary proteins for general activity and germ cell-specific processes during spermatogenesis. Abnormal sperm-nucleoprotein transition increases the instability of sperm DNA, making it more susceptible to external damage, resulting in decreased fertilization ability and miscarriage.^[10,11]^ The integrity of sperm chromatin is, therefore, now recognized as an important factor in male fertility, especially the contribution of sperm to early embryonic development. Existing studies have shown that the structure and stability of sperm chromatin are related to male fertility.^[12,13]^

In this text, we want to investigate the impact of high and low proportion of sperm histone positivity in semen sample on embryonic development, especially the results of in vitro fertilization and intracytoplasmic sperm injection. In this context, we screened and identified 1312 couples who received in vitro fertilization (IVF)/ intracytoplasmic sperm injection (ICSI) for the first time in our hospital. The differences in semen parameters, fertilization rate, normal fertilization rate, 2PN division rate, high-quality embryo rate, blastocyst formation rate, available blastocyst rate, and pregnancy outcomes were analyzed and compared.

## 2. Material and methods

### 2.1. Study design

A retrospective study was carried out on infertile couples undergoing IVF/ICSI treatments for the first time in the Center of Reproductive Medicine, First Hospital of Jilin University, Changchun, China, from January 2016 to December 2021. The ethics committee of the First Hospital of Jilin University approved the study.

**Figure 1.**
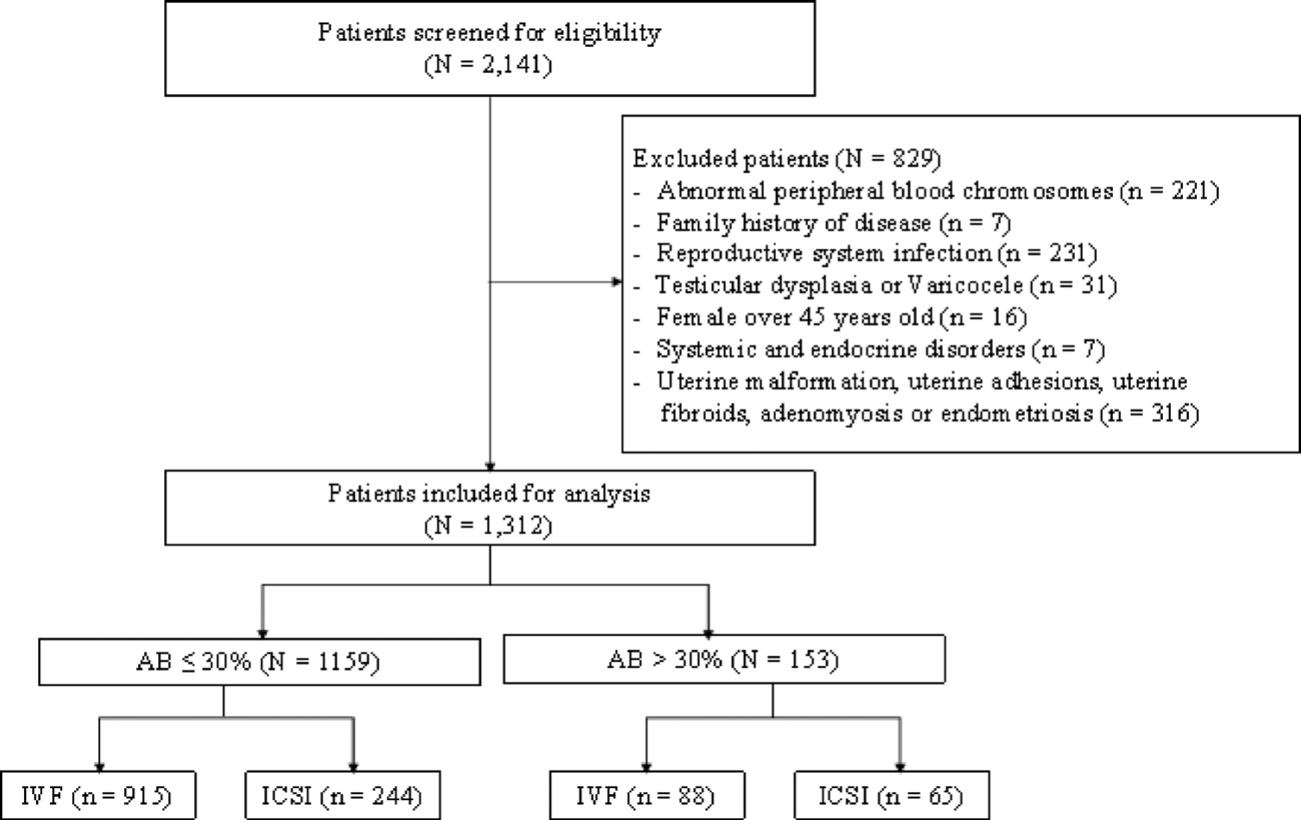
The flowchart of the study. AB = aniline blue staining positive rate.

Between January 2016 and December 2021, 2141 couples underwent IVF/ICSI treatment for the first time at our center and underwent sperm-nucleoprotein transition testing. The male patients had no family history, normal sexual function, no reproductive system infection, no testicular dysplasia and varicocele, no systemic and endocrine disease, and normal peripheral blood chromosomes. The man had a sperm nucleoprotein test within 3 months before assisted reproductive therapy. At the same time, his spouse had normal chromosomes in peripheral blood, no reproductive system infection, no systemic and endocrine system diseases, no uterine malformation, no intrauterine adhesions, no uterine fibroids, and no adenomyosis and endometriosis. Ovarian stimulation regimens vary from individual to individual, and couples with women between the ages of 20 and 45 were included in this study. Of the 2141 couples who agreed to participate in the study, 829 were excluded, and the remaining 1312 were included. Among them, 1159 couples were in the normal group of sperm-nucleoprotein transition, and 153 couples were in the abnormal group of sperm-nucleoprotein transition. The flowchart of the study is depicted in Figure 1.

### 2.2. Semen analysis

Male patients abstain from sex for 3 to 7 days, and semen was obtained by masturbation or ejaculation and collected in sterile ejaculation cups. The semen was liquefied in a 37°C water bath, the liquefaction time and the volume of the semen were recorded, and the pH value of the semen was detected. The sperm concentration and motility were calculated by a computer-assisted semen analysis system (Beijing Weili WLJY-9000).

### 2.3. Sperm nucleoprotein analysis

**Figure 2.**
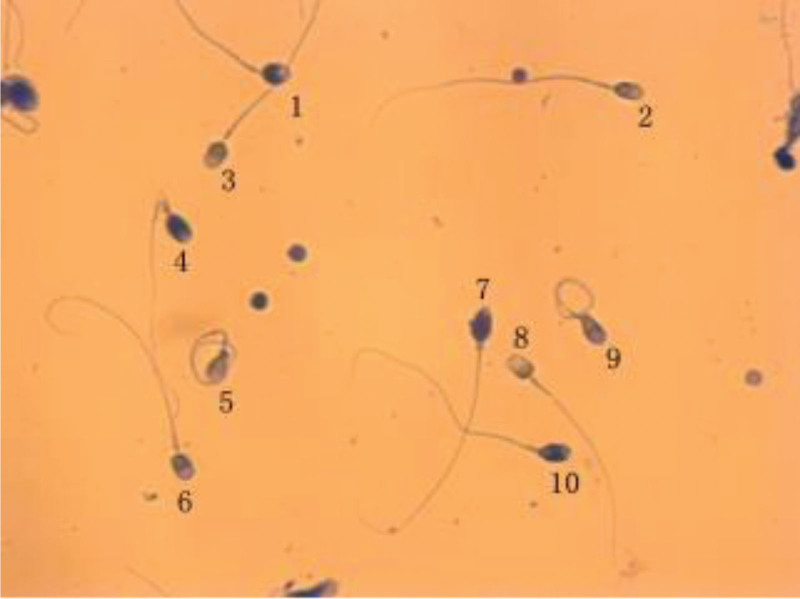
The results of sperm aniline blue staining. Positive: 1, 4, 7, 10. Negative: 2, 3, 5, 6, 8.

An aniline blue staining kit determined sperm nucleoproteins. According to the operation instructions (Shenzhen Huakang Biomedical Engineering Co., Ltd.), the reference value of abnormal sperm histones positive rate is ≤30%. The number of head-colored spermatozoa among 200 spermatozoa was counted, and the percentage was calculated. If the sperm concentration of the sample is too low, the sperm concentration can be adjusted by washing and concentration. The sperm nucleus is strongly positive if it is obviously blue, and the sperm is not stained or extremely light blue is negative. Because there is a histone-protamine transition period in sperm nucleoprotein, a few sperm are stained shallowly. If the staining degree is more than twice the average background color of negative sperm in the film (or normal control sperm), it can be counted as positive sperm. Local blue staining of sperm nucleus should also be judged as positive. The results of sperm aniline blue staining are shown in Figure 2.

### 2.4. Outcomes

To determine the IVF/ICSI embryo development, the fertilization rate, normal fertilization rate, 2PN cleavage rate, good-quality embryo rate, blastocyst formation rate, and available embryo rate were compared. To determine the assisted reproductive technology (ART) outcomes, the pregnancy rate, biochemical pregnancy rate, clinical pregnancy rate, early miscarriage rate and live birth rate were compared. Fertilization rate refers to the proportion of fertilized oocytes in the inseminated oocytes. Normal fertilization rate refers to the proportion of 2PN fertilized oocytes in the inseminated oocytes. The cleavage rate of 2PN refers to the proportion of 2PN cleaved zygotes in 2PN fertilized oocytes. Good quality embryo rate refers to the proportion of good quality embryos in cleaved zygotes. Being a “good quality embryo” requires meeting 2 criteria: first, 6 to 10 cells in the embryo on day 3; second, the embryo’s appearance under a high-power microscope reaches grade 1 or grade 2 on day 3. Blastocyst formation rate refers to the proportion of blastocysts formed in the cultured blastocysts. Available blastocyst rate refers to the proportion of blastocysts that are available for transfer in the blastocysts formed. The pregnancy rate refers to the proportion of positive pregnancies in the total embryo transfer cycles. The biochemical pregnancy rate refers to the proportion of biochemical pregnancies in the total embryo transfer cycles. Biochemical pregnancy was defined as a transient increase in serum HCG followed by a rapid decline to normal levels without morphological evidence of pregnancy on ultrasonography. Clinical pregnancy rate refers to the proportion of clinical pregnancies in total embryo transfer cycles. Clinical pregnancy was defined as the ultrasound observation of the gestational sac(s) 4 weeks after embryo transfer. The early miscarriage rate refers to the proportion of early miscarriages in clinical pregnancies. An early miscarriage indicates a pregnancy termination before 12 gestational weeks. The live birth rate refers to the proportion of live births to embryo transfer cycles. 1312 couples performed a total of 1506 frozen embryo transfer cycle.

### 2.5. Statistical analysis

SPSS software, version 23.0 (IBM, Armonk, NY) was used for statistical analysis. All numeric data are presented as the mean value ± standard deviation. Data were tested for normality using the Kolmogorov–Smirnov tests. Data complying with the normal distribution were compared by Student *t* test, while those that did not were compared by Mann–Whitney *U* test. For categorical variables, data were expressed as numbers and percentages of the total and compared by Pearson Chi-square test. With a 2-sided *P* value <.05, the statistical difference had significance.

## 3. Results

A total of 1312 infertile couples were analyzed for the study group. Baseline characteristics according to the ART procedures and sperm histones positive rate are depicted in Table 1. There were no statistical differences in’ infertility years, age, and body mass index. In men, no statistical differences were founded in liquefaction time, volume, concentration, pH, and motility of semen. In addition, women had no statistically significant differences in basal FSH, LH, or estradiol.

**Table 1:** Baseline characteristic according to sperm-nucleoprotein transition and ART procedure.

	IVF		*P* value	ICSI		*P* value
	AB ≤ 30% (n = 915)	AB > 30% (n = 88)		AB ≤ 30% (n = 244)	AB > 30% (n = 65)	
**Data on patients**						
Male age	33.46 ± 4.94	33.44 ± 5.33	.972	33.78 ± 5.19	33.20 ± 5.01	.783
Female age	32.12 ± 4.26	32.14 ± 4.90	.888	32.19 ± 4.65	31.43 ± 4.99	.366
Male body mass index (kg/m^2^)	25.82 ± 7.24	25.44 ± 3.95	.482	26.56 ± 16.3	25.29 ± 3.81	.648
Female body mass index (kg/m^2^)	22.95 ± 3.62	22.93 ± 3.72	.876	23.07 ± 3.68	23.29 ± 3.61	.564
Infertility duration (yr)	4.24 ± 3.18	3.99 ± 3.01	.596	4.03 ± 3.06	4.65 ± 3.60	.219
**Data on sperm (one semen sample per patient**)						
Sperm liquefaction time (min)	31.08 ± 13.23	30.97 ± 14.10	.599	31.57 ± 12.08	32.77 ± 14.36	.904
Sperm volume (mL)	3.38 ± 1.59	3.33 ± 1.63	.601	3.44 ± 1.44	3.47 ± 1.89	.500
Sperm concentration (million/mL)	61.31 ± 40.46	57.45 ± 38.95	.368	31.76 ± 35.66	25.86 ± 31.26	.184
Sperm pH	7.53 ± 0.13	7.54 ± 0.16	.615	7.52 ± 0.21	7.55 ± 0.15	.272
Sperm motility (a + b, %)	46.04 ± 20.16	44.79 ± 18.72	.600	23.51 ± 17.4	21.01 ± 15.97	.374
**Data on hormones**						
Female basal FSH (mIU/L)	6.62 ± 3.61	6.56 ± 2.15	.719	6.7 ± 3.54	6.79 ± 2.24	.325
Female basal LH (mIU/L)	6.06 ± 4.50	5.89 ± 3.31	.719	5.7 ± 3.46	5.09 ± 3.28	.157
Female basal estradiol (pg/mL)	55.07 ± 62.40	51.78 ± 40.51	.770	57.36 ± 77.79	48.98 ± 42.95	.642

Data are presented as mean ± standard deviation.

AB = aniline blue, ART = assisted reproductive technology, FSH=follicle-stimulating hormone, ICSI = intracytoplasmic sperm injection, IVF = in vitro fertilization, LH=luteinizing hormone.

Table 2 shows the cycle characteristics and laboratory outcomes grouped by the ART procedure and sperm histones positive rates. The number of retrieved oocytes, MII oocytes, 2PN oocytes, cleaved embryos, and good-quality day 3 embryos were similar. There were no statistical significances when mature oocyte rate, fertilization rate, normal fertilization rate, cleavage rate of 2PN, good-quality embryo rate, blastocyst formation rate, and available blastocyst rate were compared in IVF procedure. In the ICSI procedure, fertilization rate, normal fertilization rate in sperm histones normal group were higher than sperm-nucleoprotein transition abnormal group (83.19% vs 77.39%, *P* < .001; 78.76% vs 72.32%, *P* < .001). In the ICSI procedure, the cleavage rate of 2PN, good-quality embryo rate, and blastocyst formation rate were no statistically significant differences.

**Table 2:** Comparison of the laboratory outcomes of IVF/ICSI embryo transfer cycles.

	IVF		*P* value	ICSI		*P* value
	AB ≤ 30% (n = 915)	AB > 30% (n = 88)		AB ≤ 30% (n = 244)	AB > 30% (n = 65)	
No. of oocytes retrieved	12.24 ± 6.92	6.75 ± 6.75	.461	12.07 ± 8.05	12.71 ± 6.72	.309
No. of MII oocytes	10.51 ± 6.24	5.98 ± 5.98	.582	10 ± 6.84	10.62 ± 5.54	.191
No. of 2PN oocytes	6.97 ± 4.52	7.31 ± 4.70	.558	7.87 ± 5.74	7.68 ± 4.78	.849
No. of cleaved embryos	9.16 ± 5.73	5.58 ± 5.58	.710	8.36 ± 6.08	8.28 ± 4.89	.697
No. of good-quality embryos on day 3	3.47 ± 2.87	2.94 ± 2.94	.234	3.97 ± 3.90	3.69 ± 3.20	.900
Mature oocyte rate, n (%)	9621 (85.93)	952 (84.85)	.321	2439 (82.85)	690 (83.54)	.642
Fertilization rate, n (%) (fertilized oocytes/inseminated oocytes)	8622 (77.01)	843 (75.13)	.156	2029 (83.19)	534 (77.39)	<.001
Normal fertilization rate, n (%) (2PN fertilized oocytes/inseminated oocytes)	6378 (56.97)	643 (57.31)	.826	1921 (78.76)	499 (72.32)	<.001
Cleavage rate of 2PN oocytes, n (%) (2PN cleaved zygotes/2PN fertilized oocytes)	6254 (98.06)	631 (98.13)	.891	1893 (98.54)	491 (98.40)	.811
Good-quality embryo rate, n (%) (good quality embryos/cleaved zygotes)	3171 (37.83)	337 (41.00)	.074	969 (47.48)	240 (44.61)	.236
Blastocyst formation rate, n (%) (blastocysts/cultured blastocyst)	2311 (54.10)	229 (55.99)	.463	552 (54.71)	140 (51.47)	.342
Available blastocyst rate, n (%) (available blastocysts/blastocysts)	1559 (67.46)	154 (67.25)	.948	349 (63.22)	91 (65.00)	.697

Data are presented as mean ± standard deviation or numbers (percentages).

2PN = 2 pronuclei, AB = aniline blue, ICSI = intracytoplasmic sperm injection, IVF = in vitro fertilization, MII = metaphase II.

To understand the proportion of sperm histones in IVF/ICSI-assisted reproductive outcomes, we compared the primary outcomes of embryo transfer. Pregnancy outcomes following frozen embryo transfer are displayed in Table 3. There were no statistical significances when pregnancy rate, biochemical pregnancy rate, clinical pregnancy rate, early miscarriage rate, and live birth rate were compared.

**Table 3:** Comparison of the pregnancy outcomes of IVF/ICSI embryo transfer cycles.

	IVF		*P* value	ICSI		*P* value
	AB ≤ 30% (n = 915)	AB > 30% (n = 88)		AB ≤ 30% (n = 244)	AB > 30% (n = 65)	
No. of embryos transferred, n (%)			.101			.016
Single	237 (22.05)	15 (15.00)		42 (16.28)	21 (28.77)	
Double	838 (77.95)	85 (85.00)		216 (83.72)	52 (71.23)	
Embryo stage at transfer, n (%)			.635			.522
Cleavage	673 (62.60)	65 (65.00)		190 (73.64)	51 (69.86)	
Blastocyst	402 (37.40)	35 (35.00)		68 (26.36)	22 (30.14)	
Pregnancy rate, n (%) (positive beta HCG/embryo transfer cycles)	686 (63.81)	67 (67.00)	.525	171 (66.28)	47 (64.38)	.763
Biochemical pregnancy rate, n (%) (biochemical pregnancies/embryo transfer cycles)	114 (10.60)	15 (15.00)	.179	24 (9.30)	8 (10.96)	.672
Clinical pregnancy rate, n (%) (clinical pregnancies/embryo transfer cycles)	572 (53.21)	52 (52.00)	.817	147 (56.98)	39 (53.42)	.589
Early miscarriage rate, n (%) (early miscarriages/clinical pregnancies)	75 (14.26)	7 (14.89)	.905	24 (17.39)	8 (22.22)	.505
Live birth rate, n (%) (live births/embryo transfer cycles)	420 (40.82)	37 (38.95)	.723	110 (44.18)	25 (35.71)	.205

Data are presented as numbers (percentages).

AB = aniline blue, ICSI = intracytoplasmic sperm injection, IVF = in vitro fertilization.

## 4. Discussion

In our study, sperm histones positive rate may associate with the fertilization rate of ICSI. When the sperm histones positive rate was higher than the reference value, there was a negative impact on the ICSI fertilization rate, but similar results were not seen in IVF procedures. Possibly due to the different ways of fertilization in the 2 procedures, sperm with normal sperm nucleoprotein conversion is more likely to enter the oocyte during IVF. ICSI technology escapes the process of natural selection and may negatively impact the health of offspring. It is worth noting that abnormal sperm will affect the quality of embryos beyond the repair capacity of eggs and early embryos.

There is now substantial evidence supporting the conclusion that sperm chromatin integrity is important not only for fertilization but also for normal embryonic development. Many studies show sperm DNA damage affects fertilization rates, embryonic development quality, and pregnancy rates.^[14,15]^ A study of the relationship between human sperm protamine, DNA damage, and assisted reproductive outcomes has shown that sperm DNA fragmentation is associated with abnormal protein binding and results in lower fertilization rates, lower embryo quality, and lower pregnancy rates.^[12]^

Our results showed that sperm-nucleoprotein transition efficiency did not produce statistically significant differences among the indicators of IVF procedures. However, the abnormal sperm-nucleoprotein transition negatively impacted ICSI results, mainly reducing the fertilization and normal fertilization rate of ICSI procedures. It is worth noting that the effect on embryo cleavage rate and blastocyst formation rate was not statistically significant. However, due to abnormal sperm-nucleoprotein transition, the number of normally fertilized embryos decreased, resulting in a relative decrease in the number of available embryos. We did not find a significant relationship between abnormal sperm-nucleoprotein transition and clinical pregnancy and delivery, a result consistent with previous studies. However, it may also be due to the number of abnormal patients included, requiring an expansion of the number of patients in the abnormal group.

There is still some controversy about the relationship between sperm-nucleoprotein transition and assisted reproductive outcomes. Many factors influence its predictive value in assisted reproductive technology. The most important of these is that eggs and early embryos can repair sperm DNA, but this repair ability is limited. When the abnormal sperm nucleoprotein reaches a certain level and cannot be repaired, it will affect the quality of the embryo. The detection of sperm nucleoprotein transition can be used as an indicator to understand sperm quality, and it is of a certain value to evaluate sperm nucleoprotein transition before ART.

## 5. Conclusion

According to our research, an abnormal proportion of sperm histone may affect sperm function. In the ICSI procedure described in this study, the fertilization rate and 2PN fertilization rate in the normal sperm histones positive rate group were significantly higher than those in the abnormal sperm-nucleoprotein transition group, and the difference was statistically significant. However, the effect on IVF procedures was not statistically different. In addition, as a retrospective study, the number of related cases in the abnormal group is small and the overall data is scattered. This may cause some bias and limitations to the research, which needs further research in the future. After that, the next step will be to investigate the cause of abnormal changes in sperm histone to propose a treatment method that will improve the quality of the sperm nucleus and increase the chance of pregnancy.

## Author contributions

**Data curation:** Mingyue Wang, Haibo Zhu, Yuting Jiang, Ruizhi Liu

**Formal analysis:** Mingyue Wang, Haibo Zhu.

**Funding acquisition:** Ruixue Wang.

**Investigation:** Mingyue Wang, Haibo Zhu, Ruixue Wang.

**Project administration:** Ruixue Wang.

**Resources:** Ruixue Wang.

**Software:** Mingyue Wang.

**Supervision:** Ruixue Wang.

**Validation:** Mingyue Wang, Ruixue Wang.

**Writing – original draft:** Mingyue Wang.

**Writing – review & editing:** Mingyue Wang, Ruizhi Liu, Ruixue Wang.
